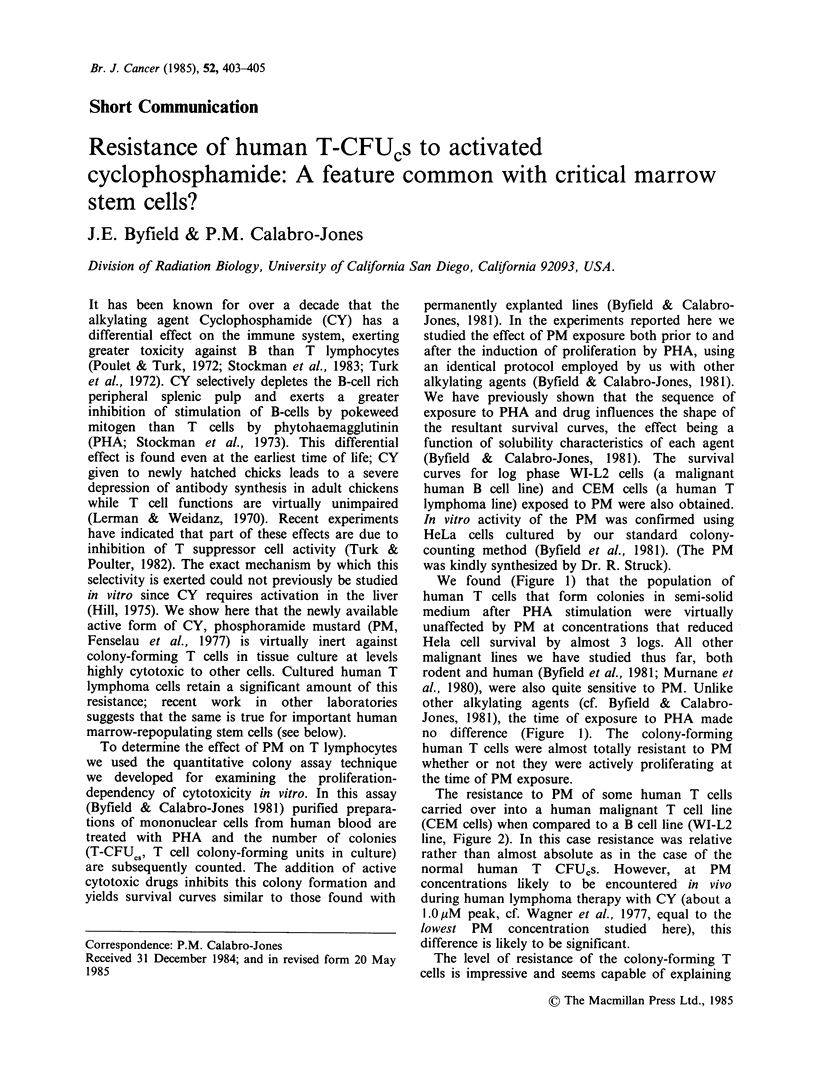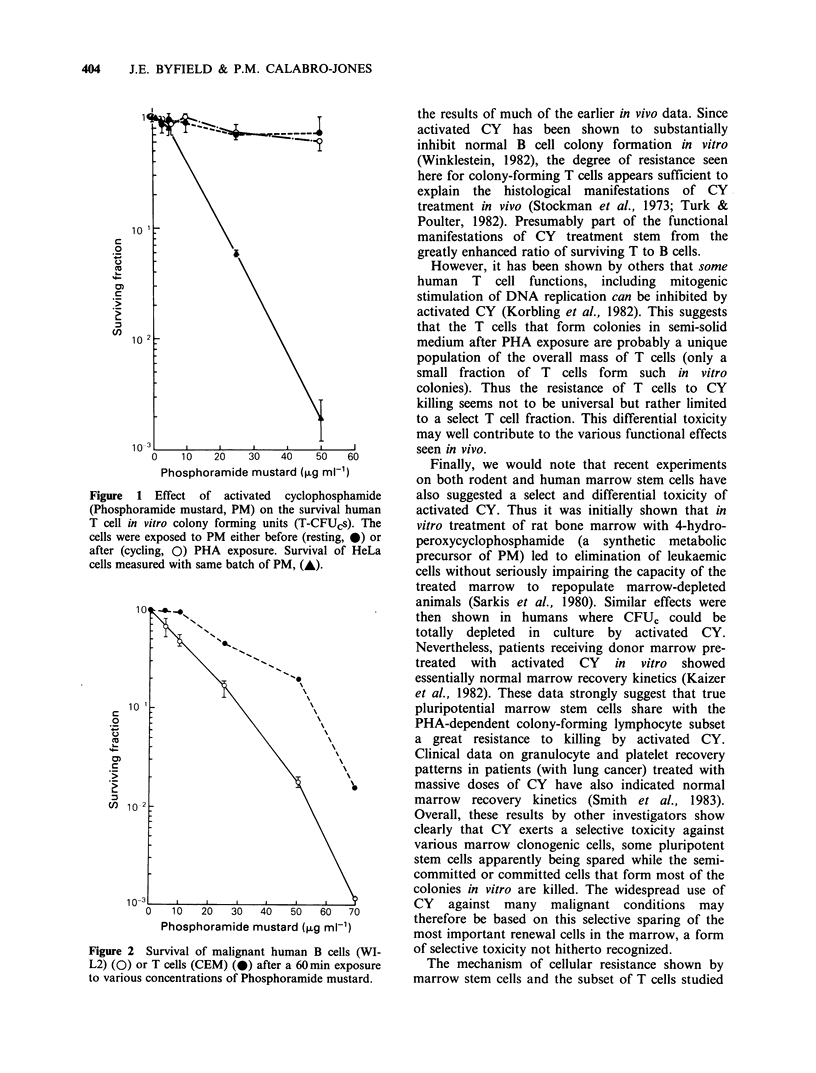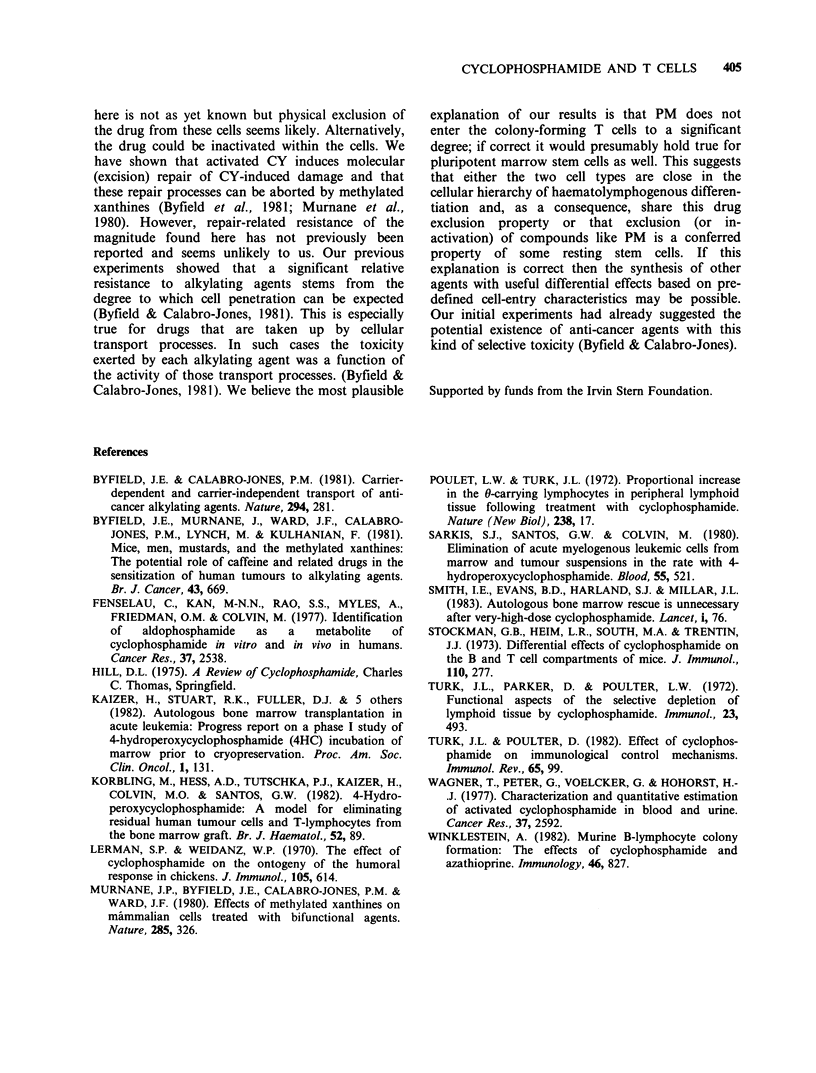# Resistance of human T-CFUcs to activated cyclophosphamide: a feature common with critical marrow stem cells?

**DOI:** 10.1038/bjc.1985.208

**Published:** 1985-09

**Authors:** J. E. Byfield, P. M. Calabro-Jones


*Br. J. Cancer* (1985), **52**, 403–405

### Short Communication

### Resistance of human T-CFU$_c$s to activated cyclophosphamide: A feature common with critical marrow stem cells?

J.E. Byfield & P.M. Calabro-Jones

*Division of Radiation Biology, University of California San Diego, California 92093, USA.*

It has been known for over a decade that the alkylating agent Cyclophosphamide (CY) has a differential effect on the immune system, exerting greater toxicity against B than T lymphocytes (Poulet & Turk, 1972; Stockman *et al.*, 1983; Turk *et al.*, 1972). CY selectively depletes the B-cell rich peripheral splenic pulp and exerts a greater inhibition of stimulation of B-cells by pokeweed mitogen than T cells by phytohaemagglutinin (PHA; Stockman *et al.*, 1973). This differential effect is found even at the earliest time of life; CY given to newly hatched chicks leads to a severe depression of antibody synthesis in adult chickens while T cell functions are virtually unimpaired (Lerman & Weidanz, 1970). Recent experiments have indicated that part of these effects are due to inhibition of T suppressor cell activity (Turk & Poulter, 1982). The exact mechanism by which this selectivity is exerted could not previously be studied *in vitro* since CY requires activation in the liver (Hill, 1975). We show here that the newly available active form of CY, phosphoramide mustard (PM, Fenselau *et al.*, 1977) is virtually inert against colony-forming T cells in tissue culture at levels highly cytotoxic to other cells. Cultured human T lymphoma cells retain a significant amount of this resistance; recent work in other laboratories suggests that the same is true for important human marrow-repopulating stem cells (see below).

To determine the effect of PM on T lymphocytes we used the quantitative colony assay technique we developed for examining the proliferation-dependency of cytotoxicity *in vitro*. In this assay (Byfield & Calabro-Jones 1981) purified preparations of mononuclear cells from human blood are treated with PHA and the number of colonies (T-CFU$_{cs}$, T cell colony-forming units in culture) are subsequently counted. The addition of active cytotoxic drugs inhibits this colony formation and yields survival curves similar to those found with permanently explanted lines (Byfield & Calabro-Jones, 1981). In the experiments reported here we studied the effect of PM exposure both prior to and after the induction of proliferation by PHA, using an identical protocol employed by us with other alkylating agents (Byfield & Calabro-Jones, 1981). We have previously shown that the sequence of exposure to PHA and drug influences the shape of the resultant survival curves, the effect being a function of solubility characteristics of each agent (Byfield & Calabro-Jones, 1981). The survival curves for log phase WI-L2 cells (a malignant human B cell line) and CEM cells (a human T lymphoma line) exposed to PM were also obtained. *In vitro* activity of the PM was confirmed using HeLa cells cultured by our standard colony-counting method (Byfield *et al.*, 1981). (The PM was kindly synthesized by Dr. R. Struck).

We found (Figure 1) that the population of human T cells that form colonies in semi-solid medium after PHA stimulation were virtually unaffected by PM at concentrations that reduced Hela cell survival by almost 3 logs. All other malignant lines we have studied thus far, both rodent and human (Byfield *et al.*, 1981; Murnane *et al.*, 1980), were also quite sensitive to PM. Unlike other alkylating agents (cf. Byfield & Calabro-Jones, 1981), the time of exposure to PHA made no difference (Figure 1). The colony-forming human T cells were almost totally resistant to PM whether or not they were actively proliferating at the time of PM exposure.

The resistance to PM of some human T cells carried over into a human malignant T cell line (CEM cells) when compared to a B cell line (WI-L2 line, Figure 2). In this case resistance was relative rather than almost absolute as in the case of the normal human T CFU$_c$s. However, at PM concentrations likely to be encountered *in vivo* during human lymphoma therapy with CY (about a 1.0 $\mu$M peak, cf. Wagner *et al.*, 1977, equal to the *lowest* PM concentration studied here), this difference is likely to be significant.

The level of resistance of the colony-forming T cells is impressive and seems capable of explaining

Correspondence: P.M. Calabro-Jones
Received 31 December 1984; and in revised form 20 May 1985

© The Macmillan Press Ltd., 1985

**Figure 1** Effect of activated cyclophosphamide (Phosphoramide mustard, PM) on the survival human T cell *in vitro* colony forming units (T-$CFU_C$s). The cells were exposed to PM either before (resting, ●) or after (cycling, ○) PHA exposure. Survival of HeLa cells measured with same batch of PM, (▲).

**Figure 2** Survival of malignant human B cells (WI-L2) (○) or T cells (CEM) (●) after a 60 min exposure to various concentrations of Phosphoramide mustard.

the results of much of the earlier *in vivo* data. Since activated CY has been shown to substantially inhibit normal B cell colony formation *in vitro* (Winklestein, 1982), the degree of resistance seen here for colony-forming T cells appears sufficient to explain the histological manifestations of CY treatment *in vivo* (Stockman *et al.*, 1973; Turk & Poulter, 1982). Presumably part of the functional manifestations of CY treatment stem from the greatly enhanced ratio of surviving T to B cells.

However, it has been shown by others that *some* human T cell functions, including mitogenic stimulation of DNA replication *can* be inhibited by activated CY (Korbling *et al.*, 1982). This suggests that the T cells that form colonies in semi-solid medium after PHA exposure are probably a unique population of the overall mass of T cells (only a small fraction of T cells form such *in vitro* colonies). Thus the resistance of T cells to CY killing seems not to be universal but rather limited to a select T cell fraction. This differential toxicity may well contribute to the various functional effects seen *in vivo*.

Finally, we would note that recent experiments on both rodent and human marrow stem cells have also suggested a select and differential toxicity of activated CY. Thus it was initially shown that *in vitro* treatment of rat bone marrow with 4-hydroperoxycyclophosphamide (a synthetic metabolic precursor of PM) led to elimination of leukaemic cells without seriously impairing the capacity of the treated marrow to repopulate marrow-depleted animals (Sarkis *et al.*, 1980). Similar effects were then shown in humans where $CFU_c$ could be totally depleted in culture by activated CY. Nevertheless, patients receiving donor marrow pretreated with activated CY *in vitro* showed essentially normal marrow recovery kinetics (Kaizer *et al.*, 1982). These data strongly suggest that true pluripotential marrow stem cells share with the PHA-dependent colony-forming lymphocyte subset a great resistance to killing by activated CY. Clinical data on granulocyte and platelet recovery patterns in patients (with lung cancer) treated with massive doses of CY have also indicated normal marrow recovery kinetics (Smith *et al.*, 1983). Overall, these results by other investigators show clearly that CY exerts a selective toxicity against various marrow clonogenic cells, some pluripotent stem cells apparently being spared while the semi-committed or committed cells that form most of the colonies *in vitro* are killed. The widespread use of CY against many malignant conditions may therefore be based on this selective sparing of the most important renewal cells in the marrow, a form of selective toxicity not hitherto recognized.

The mechanism of cellular resistance shown by marrow stem cells and the subset of T cells studied here is not as yet known but physical exclusion of the drug from these cells seems likely. Alternatively, the drug could be inactivated within the cells. We have shown that activated CY induces molecular (excision) repair of CY-induced damage and that these repair processes can be aborted by methylated xanthines (Byfield *et al.*, 1981; Murnane *et al.*, 1980). However, repair-related resistance of the magnitude found here has not previously been reported and seems unlikely to us. Our previous experiments showed that a significant relative resistance to alkylating agents stems from the degree to which cell penetration can be expected (Byfield & Calabro-Jones, 1981). This is especially true for drugs that are taken up by cellular transport processes. In such cases the toxicity exerted by each alkylating agent was a function of the activity of those transport processes. (Byfield & Calabro-Jones, 1981). We believe the most plausible explanation of our results is that PM does not enter the colony-forming T cells to a significant degree; if correct it would presumably hold true for pluripotent marrow stem cells as well. This suggests that either the two cell types are close in the cellular hierarchy of haematolymphogenous differentiation and, as a consequence, share this drug exclusion property or that exclusion (or inactivation) of compounds like PM is a conferred property of some resting stem cells. If this explanation is correct then the synthesis of other agents with useful differential effects based on predefined cell-entry characteristics may be possible. Our initial experiments had already suggested the potential existence of anti-cancer agents with this kind of selective toxicity (Byfield & Calabro-Jones).

Supported by funds from the Irvin Stern Foundation.

**References**

BYFIELD, J.E. & CALABRO-JONES, P.M. (1981). Carrier-dependent and carrier-independent transport of anti-cancer alkylating agents. *Nature*, **294**, 281.

BYFIELD, J.E., MURNANE, J., WARD, J.F., CALABRO-JONES, P.M., LYNCH, M. & KULHANIAN, F. (1981). Mice, men, mustards, and the methylated xanthines: The potential role of caffeine and related drugs in the sensitization of human tumours to alkylating agents. *Br. J. Cancer*, **43**, 669.

FENSELAU, C., KAN, M-N.N., RAO, S.S., MYLES, A., FRIEDMAN, O.M. & COLVIN, M. (1977). Identification of aldophosphamide as a metabolite of cyclophosphamide *in vitro* and *in vivo* in humans. *Cancer Res.*, **37**, 2538.

HILL, D.L. (1975). *A Review of Cyclophosphamide*, Charles C. Thomas, Springfield.

KAIZER, H., STUART, R.K., FULLER, D.J. & 5 others (1982). Autologous bone marrow transplantation in acute leukemia: Progress report on a phase I study of 4-hydroperoxycyclophosphamide (4HC) incubation of marrow prior to cryopreservation. *Proc. Am. Soc. Clin. Oncol.*, **1**, 131.

KORBLING, M., HESS, A.D., TUTSCHKA, P.J., KAIZER, H., COLVIN, M.O. & SANTOS, G.W. (1982). 4-Hydroperoxycyclophosphamide: A model for eliminating residual human tumour cells and T-lymphocytes from the bone marrow graft. *Br. J. Haematol.*, **52**, 89.

LERMAN, S.P. & WEIDANZ, W.P. (1970). The effect of cyclophosphamide on the ontogeny of the humoral response in chickens. *J. Immunol.*, **105**, 614.

MURNANE, J.P., BYFIELD, J.E., CALABRO-JONES, P.M. & WARD, J.F. (1980). Effects of methylated xanthines on mâmmalian cells treated with bifunctional agents. *Nature*, **285**, 326.

POULET, L.W. & TURK, J.L. (1972). Proportional increase in the θ-carrying lymphocytes in peripheral lymphoid tissue following treatment with cyclophosphamide. *Nature (New Biol)*, **238**, 17.

SARKIS, S.J., SANTOS, G.W. & COLVIN, M. (1980). Elimination of acute myelogenous leukemic cells from marrow and tumour suspensions in the rate with 4-hydroperoxycyclophosphamide. *Blood*, **55**, 521.

SMITH, I.E., EVANS, B.D., HARLAND, S.J. & MILLAR, J.L. (1983). Autologous bone marrow rescue is unnecessary after very-high-dose cyclophosphamide. *Lancet*, **i**, 76.

STOCKMAN, G.B., HEIM, L.R., SOUTH, M.A. & TRENTIN, J.J. (1973). Differential effects of cyclophosphamide on the B and T cell compartments of mice. *J. Immunol.*, **110**, 277.

TURK, J.L., PARKER, D. & POULTER, L.W. (1972). Functional aspects of the selective depletion of lymphoid tissue by cyclophosphamide. *Immunol.*, **23**, 493.

TURK, J.L. & POULTER, D. (1982). Effect of cyclophosphamide on immunological control mechanisms. *Immunol. Rev.*, **65**, 99.

WAGNER, T., PETER, G., VOELCKER, G. & HOHORST, H.-.J. (1977). Characterization and quantitative estimation of activated cyclophosphamide in blood and urine. *Cancer Res.*, **37**, 2592.

WINKLESTEIN, A. (1982). Murine B-lymphocyte colony formation: The effects of cyclophosphamide and azathioprine. *Immunology*, **46**, 827.